# Brain anatomy of the marine isopod *Saduria entomon* Linnaeus, 1758 (Valvifera, Isopoda) with special emphasis on the olfactory pathway

**DOI:** 10.3389/fnana.2013.00032

**Published:** 2013-10-07

**Authors:** Matthes Kenning, Steffen Harzsch

**Affiliations:** Cytologie und Evolutionsbiologie, Zoologisches Institut und Museum, Ernst Moritz Arndt Universität Greifswald, Greifswald, Germany

**Keywords:** isopoda, central nervous system, immunohistochemistry, neurophylogeny, olfaction

## Abstract

Representatives of at least six crustacean taxa managed to establish a terrestrial life style during their evolutionary history and the Oniscidea (Isopoda) are currently held as the most successfully terrestrialized malacostracan crustaceans. The brain architecture of terrestrial isopods is fairly well understood and studies on this field suggest that the evolutionary transition from sea to land in isopods coincided with a considerable size reduction and functional loss of their first pair of antennae and associated brain areas. This finding suggests that terrestrial isopods may have no or poor abilities to detect volatile substances but that their chemosensory ecology is most likely restricted to contact chemoreception. In this study, we explored how the brain of a marine isopod and particularly its olfactory system compares to that of terrestrial relatives. Using histochemical and immunohistochemical labeling, brightfield and confocal laser-scan microscopy, we show that in the marine isopod *Saduria entomon* aesthetascs on the first pair of antennae provide input to a well defined deutocerebrum (DC). The deutocerebral chemosensory lobes (DCL) are divided into spherical neuropil compartments, the olfactory glomeruli (og). Secondary processing areas in the lateral protocerebrum (lPC) are supplied by a thin but distinct projection neuron tract (PNT) with a contralateral connection. Hence, contrary to terrestrial Isopoda, *S. entomon* has at least the neuronal substrate to perceive and process olfactory stimuli suggesting the originally marine isopod lineage had olfactory abilities comparable to that of other malacostracan crustaceans.

## Introduction

**Figure 1:**
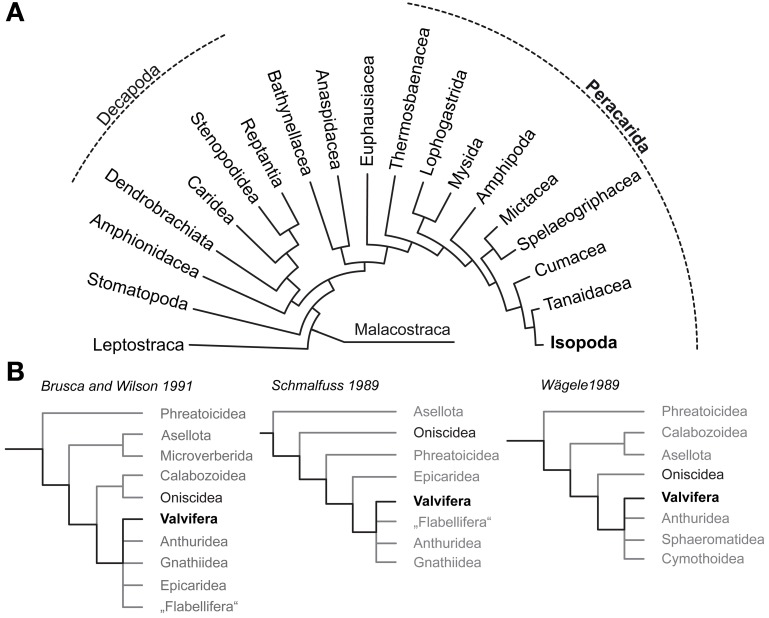
**Phylogenetic relationships of Malacostraca and Isopoda. (A)** Phylogeny of Malacostraca compiled after Richter and Scholtz (2001) **(B)** Competing hypotheses on the phylogenetic relationships within Isopoda and unresolved positioning of Valvifera according to Brusca and Wilson (1991), Schmalfuss (1989) and Wägele (1989).

The Isopoda (Peracarida; Figure 1) comprise roughly 10,000 known species, but more cryptic biotopes like the deep sea remain to be explored for isopod diversity. The body size of isopods ranges from a few hundred micrometers in the interstitial Microcerberidea to impressive 50 cm in *Bathynomus giganteus*. In their over 300 million years lasting history, with a fossil record dating back to the Carboniferous (Bandel, 1967; Wilson, 2008), the Isopoda underwent a extensive radiation and colonized almost every aquatic habitat ranging from the deepest trench to shallow shelf waters and freshwater lakes. Besides scavengers, isopods are parasites, predators, and cannibals as well as prey, but they are also known for their highly developed social behavior (Kaestner, 1993; Schmalfuss, 2003; Duffy and Thiel, 2007; Linsenmair, 2007; Schmidt, 2008). Along with the first terrestrial ancestors of Hexapoda, at least five lineages of malacostracan crustaceans independently succeeded in colonizing land (Bliss and Mantel, 1968; Powers and Bliss, 1983; Greenaway, 1988, 1999; Hartnoll, 1988). While most of these taxa are still constrained to an aquatic milieu during larval development, several representatives of Oniscidea (e.g., the xerophilic desert ispod *Hemilepistus reaumuri*) achieved a level of terrestrialness that completely released them from their marine heritage, likely making Isopoda the most successful land living crustaceans.

The evolutionary transition from marine to terrestrial environments requires a number of physiological adaptations. These relate, for example, to gas exchange, ion and water balance, excretion, thermoregulation, molting, and reproduction (Bliss and Mantel, 1968; Edney, 1968; Powers and Bliss, 1983; Burggren and McMahon, 1988; Greenaway, 1988, 1999, 2003; McMahon and Burggren, 1988). What is more, sensory organs must function in air instead of water. This has in many cases a profound impact on the morphology and physiology of visual, mechanosensory and chemosensory systems, reshaping these organs during the evolutionary colonization of the new habitat (Weissburg, 2010; Hansson et al., 2011; Hay, 2011). In general, our current knowledge of the organization of crustacean olfactory systems is heavily biased toward members of the Decapoda [e.g., crabs, crayfish clawed and spiny lobsters; reviewed by, e.g., Schachtner et al. (2005); Schmidt and Mellon (2011); Harzsch et al. (2012); Sandeman et al. (in press)]. However, studies that examined land living hermit crabs (*Birgus latro*: Krieger et al., 2010; *Coenobita clypeatus*: Harzsch and Hansson, 2008; Brown and Wolff, 2012; Polanska et al., 2012) point to a sophisticated and well adapted olfactory system in terms of antennular and neuronal morphology, suggesting that these animals were successful in establishing aerial olfaction (Hansson et al., 2011).

To answer the question why terrestrial isopods which have mastered crucial steps to cope with a life on land failed in adapting their olfactory system to function in air it is necessary to understand the chemosensory system of the ancestral isopod lineages that lived in the marine habitat. Therefore, this study sets out to enrich our knowledge on brain architecture in marine Isopoda by analyzing the neuroanatomy of the Baltic Sea glacial relict *Saduria entomon* (Valvifera) with special respect to the olfactory pathway. The phylogeny of Isopoda is still subject of intensive discussions and the Valvifera have been rooted in almost every position of the phylogenetic tree (Schmalfuss, 1989; Wägele, 1989; Brusca and Wilson, 1991; Wetzer, 2002; Schmidt, 2008; Richter et al., 2009; Wirkner and Richter, 2010; see Figure 1B) but the long-tailed morphology of *S. entomon* suggests a derived position (Brusca and Wilson, 1991). We are perfectly aware of the fact that for tracing the evolutionary transition from marine to terrestrial habitats in Isopoda, obtaining information from a more ancestral marine taxon would be preferrable. However, we are confident that the data on the brain of this *S. entomon* provided in the following is valuable in its own, and also might serve as a proxy for the plesiomorphic state of the brain architecture in marine Isopoda, nonetheless. Whereas terrestrial Isopoda have repeatedly served as models to study the olfactory pathway with respect to morphology, physiology, and behavior, our knowledge on the chemical ecology of marine isopods is close to zero [reviewed in Thiel (2011)]. Among the few studies on chemically-guided behavior in aquatic isopods that we are aware of, are those of Thompson and Manning (1981) on mate choice in the freshwater isopod *Asellus aquaticus* (Asselota) and on feeding behavior in the marine *S. entomon* (Valfivera) (Green, 1957). In the latter the antennular morphology and aesthetasc ultrastructure has been examined by Pynnönen (1985). As for behavior, the few available studies on the isopod brain architecture have focused on terrestrial representatives with the most comprehensive accounts dating back to the early 20th century (Gräber, 1933; Walker, 1935; Hanström, 1968; Alexander, 1970; Warburg and Rosenberg, 1978). Classical morphological descriptions also exist for the brains of two fully terrestrial members of the Oniscidea, *Armadillidium vulgare* (Schmitz, 1989) and *Hemilepistus reaumuri* (Kacem-Lachkar, 2000) in both of which the deutocerebral olfactory pathway has eroded away. More recently, (immuno)histochemical studies included the characterization of the optic neuropils (optN) underlying the compound eyes (Sinakevitch et al., 2003) and the localization of various neurotransmitters in the brain and ventral nerve cord (Warburg and Rosenberg, 1978; Martin and Dubois, 1981; Thompson et al., 1994; Nussbaum and Dircksen, 1995; Fouda et al., 2010; Wilcockson et al., 2011) whereas the nervous system of marine isopods remains poorly examined.

## Materials and methods

*S. entomon* has a circumpolar distribution and is abundantly found in the northern Baltic Sea (Bothnian, Åland and Archipelago Seas, and in the Gulf of Finland), the Black Sea and certain boreal freshwater lakes. The animals studied here were collected in the Tvärminne Storfjärd, Gulf of Finland (Baltic Sea) near the Tvärminne Zoological Station with permission and support of the station authorities. For immunohistochemical experiments, a total of 15 specimens of both sexes were anaesthetized in ice-cooled mineral water, decapitated and fixated in either (i) 4% paraformaldehyde (PFA) in 0.1 M phosphate buffered saline (PBS), pH 7.4 for 2 h or (ii) for 24 h in 4% zinc-paraformaldehyde (for details see Ott, 2008) and subsequently stored in PBS (i) or HEPES buffer at 4°C (ii). Vibratome sectioning and immunohistochemical labeling of the tissues followed standard protocols (see, e.g., Harzsch et al., 2011; Sombke et al., 2011; Krieger et al., 2012; Kenning et al., 2013). Whole-mount preparations were performed according to Ott (2008). Table 1 summarizes all labeling procedures and antibodies used. Immunohistochemical labeling was primarily used to visualize the neuronal and neuropil architecture. Thus, we applied a small range of antibodies, all of which are well established and known to label their respective antigens in the brains of arthropods. An overview of the specificity of the antibodies used can be found in Kenning et al. (2013). For histological preparations, Bouin fixated heads (saturated picrinic acid, formaldehyde, glacial acetic acid, 15:5:1) were dehydrated in an ascending series of ethanol, followed by a single incubation in xylene and two consecutive infiltrations of paraffin at 60°C for 1 and 2 h, respectively. The specimens were then removed and embedded in fresh paraffin. Horizontal sections were cut at a thickness of 6 μm using a microtome (Leica RM 2145), stained with Azan according to Geidies, and mounted in Roti-Histokitt (Carl Roth). Sections were digitized with a Nikon Eclipse 90i microscope equipped with a digital Nikon DS2-MBW camera. In addition, selected preparations were analyzed with a Leica SP5 II confocal laser scanning microscope. Autofluorescence microscopy was used to visualize the morphology of the first and second antenna as suggested by Haug et al. (2011). Digital images were processed with Adobe Photoshop, if necessary. Only global picture enhancement features (i.e., brightness and contrast) have been used. 3D reconstructions base on paraffin thick-section series. Alignment and reconstruction were performed with AMIRA 5.2 (Visage Imaging). In each section, contours of the neuropils were traced, out of which a 3D model was generated. For compiling the diagrams, we used Adobe Illustrator CS4. The neuroanatomical nomenclature of this manuscript is based on Sandeman et al. (1992), Richter et al. (2010), and Loesel et al. (2013) for the description of the neuropils, cell cluster, and tracts. Moreover we propose new terms to facilitate homologization of certain characters between malacostracan crustaceans and hexapods.

**Table 1:** **Primary and secondary antibodies used in the study**.

**Labeling agent**	**Dilutions and specifications**
**PRIMARY**	
Polyclonal rabbit anti-FMRFamide	1:2000; Acris/Immunostar; Cat. No. 20091; Harzsch et al., 2011; Krieger et al., 2012; Kenning et al., 2013
Monoclonal mouse anti-synapsin	1:30; SYNORF1, DSHB; Harzsch et al., 2011; Krieger et al., 2012; Kenning et al., 2013
Polyclonal rabbit anti-5-HT	1:1000; Immunostar, Cat. No. 20080, Kenning et al., 2013
Anti-tyronisated tubulin	1:1000; Sigma–Aldrich; Cat. No.T9028; Sombke et al., 2011; Kenning et al., 2013
**SECONDARY**	
Anti-rabbit AlexaFluor488	Goat anti-rabbit IgG (H + L) antibody, Invitrogen; MolecularProbes; Cat. No. A-11008
Anti-mouse Cy3	Cy3-conjugated AffiniPure goat anti-Mouse IgG (H + L) antibody, Jackson ImmunoResearch Laboratories Inc. Cat. No. 115-165-003
**NUCLEAR COUNTER STAIN**	
	0.05%, bisBenzimid H 33258, Sigma–Aldrich; Cat. No. 23491-45-4

## Results

**Figure 2:**
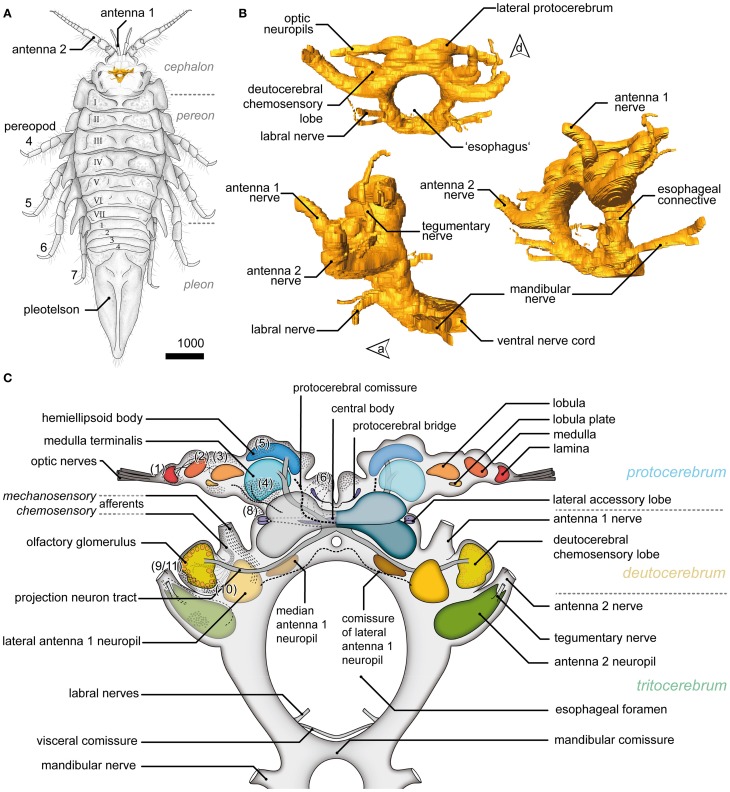
**General overview of the brain in *Saduria entomon*. (A)** A sketch of *Saduria entmon* showing the position and relative proportion of the brain. **(B)** 3D reconstruction of the brain from frontal (top left), lateral (bottom left) an ventrofrontal (right) showing the major neuromeres and sensory afferents. **(C)** Semidiagrammatic representation of *S. entomons*' brain.

**Figure 3:**
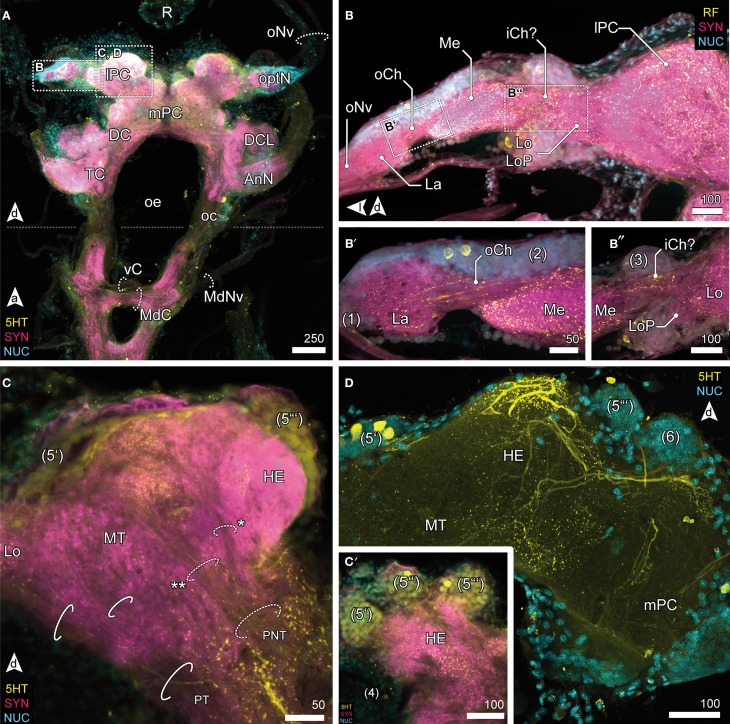
**Optic neuropils and the lateral protocerebrum. (A)** Overview of the brain from posterior and dorsal view of the anterior part of the VNC. The dotted line indicates the change of orientation between brain and nerve cord. The optN are supplied by the R by oNv. Two commissures connect the oc; an anterior vC, and the posterior MdC out of which the MdNv emerge. **(B)** Posterior view of the optN **(B′)** Magnification of connection between La and Me, the oCh. **(B″)** Magnification of connection between Me and Lo. The connectivity remains unresolved, thus the presumptive iCh is labeled with “?.” The Lo is accompanied by a small loP neuropil. **(C)** The lPC in different section planes, center **(C)** and anterior end **(C′)**. The lPC is innervated by the PNT, giving off a branch innervating the HE (asterisk), the remainder proceeds into the MT (double asterisk). The PT connects optN and MT with the mPC. **(C′)** The HE is innervated by neurons located in three bulb-like clusters (5) anterodorsal to the neuropil. **(D)** The HE is heavily innervated by 5HTir neurons located in cluster (5′), giving of fine branches into the deeper layers and MT.

**Figure 4:**
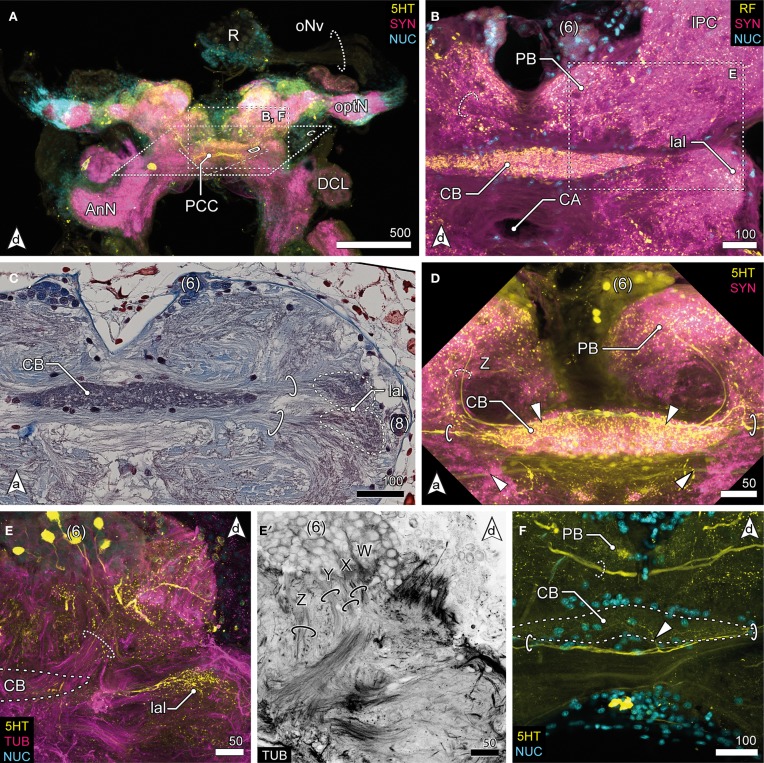
**Median protocerebrum and central complex. (A)** Overview of the brain from posterior. A large 5HTir PCC connects both hemispheric lPC ventrally to the CB. **(B)** The central complex labeled against RF-amides showing the anterodorsal cluster 6, the PB, CB and lal, frontal section. The CB is ensheathed by a couple of somata, none of which showing RFir (compare with **F**). The PB is interconnected by a commissure (dotted circle). **(C)** Ventral extensions of (6) and (8), CB and lal. A couple of somata are distributed around the CB. The bipartite lal are interconnected by two large commissures (solid circles), horizontal paraffin section. **(D)** Frontal section of CB. 5HTir of commissures interconnecting the lal (solid circles). The CB is also innervated by neurons (dotted circle, Z) from (6), corresponding to tract Z [according to Utting et al. (2000); dotted circle, see **E′]** **(E)** Anterolateral region of the mPC showing the anterior unit of the lal, also a small part of the PNT is visible (dotted circle), frontal section. The CB (posterior to this section plane) is indicated by a dotted line. **(E′)** Single channel image showing TUBir of projections of cluster 6 corresponding to tract W, X, Y and Z, innervating the CB and lal. **(F)** Frontal section of CB. 5HTir of PB commissure (dotted circle) and arborizations of neurons from (8) (arrowhead) bypassing the CB ventrally (solid circles).

The position, general appearance and a schematic of the syncerebrum of *S. entomon* is shown in Figure 2. The neuraxis is prominently bent dorsally in the region of the esophageal connectives (Figures 2B,C, dotted line in 3A), resulting in an L-shape in which the brain lies approximately perpendicular to the ventral nerve cord. All following descriptions refer to the body axis. Three neuromeres can be identified from dorsal to ventral. The brain is dominated by the dorsal most protocerebrum (PC), in particular by the protrusion of the lateral protocerebrum (lPC) and optN (Figures 2B,C, 3, 4A).

### Optic neuropils

Visual afferents from the retina (R; Figures 3A, 4A) project *via* long optic nerves (oNv, i.e., up to 13 mm in larger specimen) laterally into the brain and supply four consecutive and closely associated optN (Figures 2, 3). These are surrounded by a cell cortex comprising the cell cluster 1–3 [according to the terminology by Sandeman et al. (1992)]. The first, most distal neuropil, the lamina (La) is displaced distally away from the brain and interconnected with the second neuropil (medulla, Me) by the (outer) optic chiasm (oCh, Figures 3B,B′). The connectivity of the latter and the third neuropil (lobula, Lo) could not be clarified (iCh?; Figures 3B,B″) due its close proximity to the lPC. The lamina is weakly immunoreactive for the antibodies used. In contrast, the medulla is intensively labeled with antisera against FMRF-amides (RF), serotonin (5HT), and synapsin (SYN), yet is unstructured. The lobula is accompanied by a small fourth neuropil, the lobula plate (LoP), which only could be vizualized unequivocally by anti-synapsin labeling (LoP; Figure 3B″). Its connectivity with medulla, lobula, and lPC could not be clarified with certainty.

### Lateral protocerebrum

The lobula and LoP are closely associated with a neuropil complex comprising the lPC (Figures 2C, 3C,D). Within the lPC, no clearly demarcated neuropil regions are distinguishable. However, anti-synapsin labeling reveals an intensively stained region surmounting the lPC dorsomedially like a cap, the hemiellipsoid body (HE; Figure 3C). This neuropil is formed by neurites from neurons whose somata are located in three bulb-like and very distinct spherical clusters located dorsolaterally, dorsally and dorsomedially of the protocerebral complex (cluster 5′–5^″′^, Figure 3C′). Another neuropil of the lPC, the medulla terminalis (MT), has a homogeneous texture and is innervated by a large but indistinct posterolateral cluster (cluster 4; Figures 3C,C′). Both neuropils of the lPC are supplied by a prominent neurite bundle emerging from the deutocerebrum (DC), the projection neuron tract [PNT according to Loesel et al. (2013); olfactory globular tract according to the traditional terminology of Sandeman et al. (1992); Figure 2C, dotted circles in 3C]. This tract gives rise to a small branch innervating the hemiellipsoid bodies (asterisk in Figure 3C), the remainder could not be traced any further but most likely ends within the medulla terminalis (double asterisk in Figure 3C). A second tract (protocerebral tract, PT, solid circles; Figure 3C) connecting the lPC with the “central” brain is located laterally to the medulla terminalis. The protocerebral tract is composed of two branches, one of which terminates within the lPC whereas the other proceeds toward the optN.

### Median protocerebrum

The median protocerebrum (mPC) is easily identified in sections by the central complex that is composed of three distinct neuropils: the central body (CB), the lateral accessory lobes (lal) and the protocerebral bridge (PB) (Figures 2C, 4). Two cell clusters are associated with this complex. The unpaired CB extends transversely across the midline and provides a conspicuous cigar-shaped landmark in the median brain, just dorsal to the cerebral artery (CA) which pierces the brain in an anterior—posterior direction. It is intensively labeled by all antibodies used here but does not show any obvious subdivisions. The neuropil is completely embedded between several commissural neurite bundles (solid circles in Figures 4C,D,F). Further, it is surrounded by a number of somata which are likely glia cells (Figures 4B–D,F), as none of them exhibited immunoreactivity for the antibodies used. The neuropil is supplied by neurites emanating from a paired anterodorsal cell cluster [(6) in Figures 4B–E]. These neurites form synapses in a small paired neuropil, the PB (Figures 4B,D,F) and project in four distinct tracts (W, X, Y, Z, Figure 4E′; dotted circle in D) to the CB. Both PB neuropils are connected by a commissure that shows no immunoreactivity for FMRF-amides but contains at least one large serotonergic neurite (dotted circles in Figures 4B,F). The lateral accessory lobe, a bilaterally paired neuropil is located laterally to the CB and is innervated by neurons from cluster (6) and from a small bulb-like cluster (8) protruding laterally from the brain (Figure 4C). It is distinctly immunoreactive for 5HT but its bipartite nature is more clearly seen in histological sections, (dotted circles in Figure 4C). Two large commissures, anterior and posterior to the CB, interconnect the accessory lobes (solid circles in Figures 4C,D,F) and extend arborizations into the midline neuropil (arrowhead in Figure 4F). Anti-serotonin-labeling reveals another large commissure connecting both hemispheres of the lPC (PCC, Figure 4A).

### Deutocerebrum

**Figure 5:**
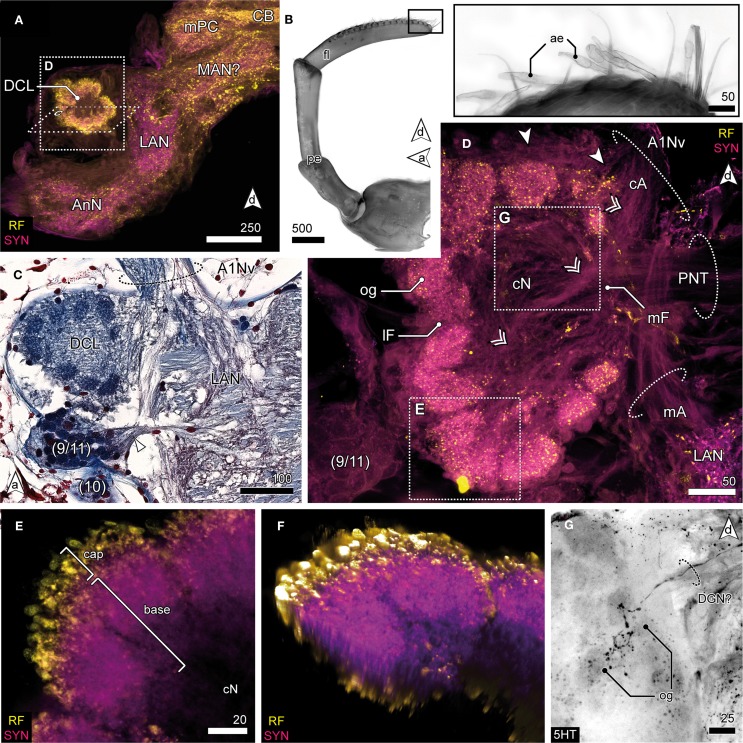
**Deutocerebrum and the deutocerebral chemosensory lobe. (A)** DC and TC as shown in anti-RF and anti-SYN labeling. The DCL protrudes laterally from the DC, flanking the LAN. **(B)** The distal antennal segment bearing several tufts composed of 3 accessory setae and 2 aesthetascs (inset) giving rise to A1Nv; UV-autofluorescence. **(C)** Horizontal paraffin section of the DC showing innervation of DCL by A1Nv (dotted circle), cluster (9/11) and (10), and the LAN. **(D)** The DCL as reveal in anti-AST and anti-SYN labeling showing its glomerular organization surrounding the cN. A1Nv sends off two branches into the DC, presumptive chemosensory afferents (cA) innervate the og from the periphery (arrowhead) but also from the center (double arrowhead). Another branch proceeds toward the LAN and carries mechanosensory afferents (mA) and probably also efferents. A cluster of local interneurons (9/11) is located posteriolateral to the DCL and sends out neurites into the DCL through a lateral foramen (lF; not visible in this section plane). Output is provided by projection neurons (PNT) through a medial foramen (mF). **(E)** Close up of a single glomerulus illustrating the subdivision into a cap region showing intensive neuropeptide-ir and a base with a predominant anti-SYN labeling. **(F)** Volume rendering of image-stack shown in **(E)**, showing that the center of the glomerulus is devoid of neuropeptides-ir while the periphery is intensively stained. **(G)** 5HTir of the DCL's surface showing a single prominent serotonergic neurite with multiglomerular innervations.

The DC, though small in proportions, is characterized by distinctive lateral outswellings comprising the deutocerebral chemosensory lobes (DCL; olfactory neuropils or olfactory lobes according to the traditional terminology; Figures 2, 5). This nearly spherical neuropil of approximately 250 μm in diameter protrudes laterally from the brain and is thus easily detached during the preparation procedure. From section series, we estimate that it consists of about 80 small, distinct neuropil subunits of roughly spherical shape, the olfactory glomeruli (og, Figures 2C, 5C–G). The glomeruli are arranged radially around the periphery of the DCL and surround a coarse neuropil (cN) of loose neuronal processes. They are further divided into two distinct domains, a distal cap and proximal base. The cap is strongly labeled by the antisera against neuropeptides whereas in the base, SYN immunoreactivity predominates (Figures 5E,F). The DCL is supplied by afferents from aesthetascs situated on the distal segment of the antenna 1 (Figure 5B) *via* the antenna 1 nerve (A1Nv, Figures 2C, 5C,D). Afferents (cA) enter the chemosensory lobes' og from the periphery (arrowheads in Figure 5D) and apparently also from within the lobe (double arrowheads in Figure 5D) through a median foramen (mF). The latter is a quite remarkable finding that requires further investigation. A small cell cluster (9/11) is located ventrolaterally and houses local interneurons whose neurites enter the neuropil between two glomeruli through a lateral foramen (lF, Figure 5D). A posterioventral extension of this cluster also innervates medial parts of the DC (arrowhead in Figure 5C). 5HT labeling reveals a single prominent serotonergic neurite innervating several og, yet, the corresponding soma has not been found (Figure 5G). Output from the DCL is provided by a large tract of projection neurons (PNT) emerging from the center of the neuropil through the mF (Figures 2C, 5C,D). The corresponding somata are located in a small irregularly shaped cluster (10) posteroventrally to the DCL (Figure 5C). The PNT proceeds further into the mPC to target neuropils of the lPC (Figures 2C, 3C). At the level of and slightly posterior to the CB, both hemispheric branches of the PNT approach the midline and form a chiasm in which several neurites enter the contralateral side (Stemme and Eickhoff, pers. commun.). Apart from the presumptive chemosensory afferents entering the DCL, the antenna 1 nerve gives rise to another branch (mA) dorsomedially that proceeds further into the DC innervating the lateral antenna 1 neuropil (LAN), an undivided paired neuropil of inconspicuous shape located medially to the DCL (Figures 2C, 5A,C,D). These neuropils are interconnected by a thin commissure (not shown). Between the lateral antenna 1 neuropil and CB, a diffuse bilaterally paired neuropil region is visible showing an irregular immunoreactive patterning. Its location suggests that it may constitute the median antenna 1 neuropil (MAN), although it is not clear from our specimens whether this region actually receives any input from antenna 1 (Figures 2C, 5A).

### Tritocerebrum

**Figure 6:**
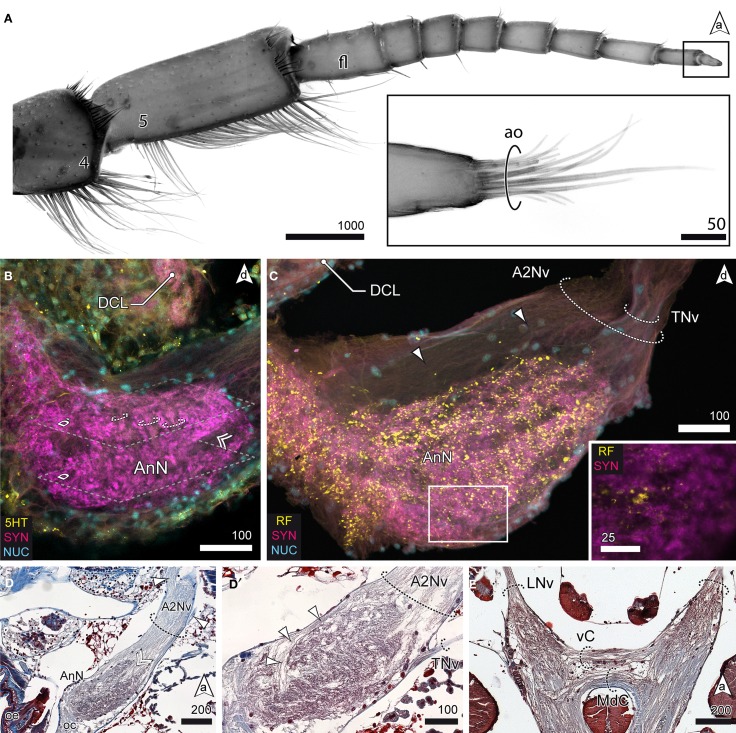
**The tritocerebrum. (A)** UV-autofluorescence of antenna 2 and the tip showing the last two antennal segments and fl bearing the ao (inset). **(B)** AnN as revealed in anti-5HT and anti-SYN labeling. Parts of A2Nv enter the neuropil in a groove like depression (double arrowhead, compare with **D**). The AnN shows traces of repetitively arranged neurite bundles crossing the neuropil in a perpendicular manner (dotted circles). **(C,D′)** The TNv projects into the AnN posteriolaterally while parts of the A2Nv bypass the neuropil anterodorsally and the target domains in the posterior parts of the neuropil. SYNir illustrates the microglomerular organization of the AnN (inset **C**). **(D)** Nerves emerging from A2Nv target muscles at the base of antenna 2 (arrowheads). Parts of A2Nv enter the neuropil in a groove like depression (double arrowhead). **(E)** Anterior part of the VNC showing two commissures connecting the oc. An anterior vC giving rise to LNv and the posterior MdC.

Aside the protocerebral neuropils, the brain of *S. entomon* is dominated by the tritocerebral neuropils protruding anterolaterally from the esophageal connectives and targeted by a large nerve that is supplied by receptors on antenna 2, including the apical cone (Figures 2C, 6A). The nerve is mainly associated with the antenna directly but a smaller lateral branch can be traced to muscles at the base of antenna 2 (arrowheads in Figure 6D). Close to the esophageal foramen the nerve thickens and enlarges to the distinct spindle-shaped antenna 2 neuropil (AnN, Figures 2B,C, 3A, 6B–D). This neuropil is labeled by all antibodies used. In particular anti-synapsin labeling reveals a rather complex structure reminiscent of a microglomerular organization (inset, Figure 6C). In addition, in parts the neuropil displays a transverse striation (dotted circles in Figure 6B) although the current data is not conclusive. The area where parts of the A2Nv enter the neuropil, i.e., the tip, appears to be grooved or sunken in (double arrowhead in Figures 6B,D). A small branch of the antenna 2 nerve bypasses the antenna 2 neuropil dorsomedially (arrowheads in Figures 6C,D′). It emerges from the medial region of the antenna 2 neuropil, close to the esophageal connectives. A thin nerve (tegumental nerve, TNv; Figures 2B,C, 6C,D′) emerges from the antenna 2 neuropil posteriorly and ascends dorsally, bypassing the optN. The esophageal connectives are interconnected by two commissures ventrally to the tritocerebral domain that merges with the ventral nerve cord. The anterior visceral commissure (vC) is thin, gives rise to the labral nerves (LNv) anteroventrally and is located directly anteriorly to the larger mandibular commissure (MdC, Figures 3A, 6E).

## Discussion

The general morphology of the brain in *S. entomon* presented above broadly equals what has been reported previously for other representatives of the Isopoda. However, the most pervasive difference is found in the peripheral and central olfactory pathway when compared with terrestrial representatives.

### Antenna 1

Malacostracan Crustacea are typically equipped with two pairs of antennae, a first pair (antennule or antenna 1) associated with the DC, and a second pair (antenna or antenna 2) associated with the tritocerebrum. In addition to bimodal chemo- and mechanosensilla distributed along the length of the antenna 1, the distal segment of the first antennae bears an array of specialized chemoreceptive sensilla housing the branched dendrites of olfactory sensory neurons, the aesthetascs (Hallberg et al., 1992, 1997; Hallberg and Hansson, 1999; Hallberg and Skog, 2011; Schmidt and Mellon, 2011). As shown in previous studies (Kovalevskij, 1864; Pynnönen, 1985), *S. entomon* possesses well developed antennulae, with their distal segment equipped with aesthetascs. Although sparsely innervated by dendritic processes, their general morphology strongly resembles those of other marine crustaceans [e.g., *Panulirus argus*: (Laverack, 1964); *Homarus americanus*: (Shelton and Laverack, 1970); *Idotea baltica*: (Guse, 1983); reviewed in Hallberg and Skog (2011)], suggestive of a chemoreceptive function. They are located in a row of up to 30 tufts on the ventral edge of antenna 1 pointing anteriorly, with each tuft bearing a pair of aesthetascs that is accompanied by three accessory setae (Pynnönen, 1985). Thus, with approximately 60 aesthetascs, *S. entomon* has a relatively low number compared to other marine malacostracans but is still undercut, e.g., by the brachyuran *Uca pugnax* (Brachyura, Decapoda) with only about 26 aesthetascs (Beltz et al., 2003). At the other end of the range *Panulirus interuptus* (Achelata, Decapoda) was determined to house up to 1786 aesthetascs per antenna 1 (Beltz et al., 2003). When dealing with such numbers, it has to be noted that during the course of a crustaceans' life aesthetascs are continuously added while the animal grows (Beltz et al., 2003). In contrast, oniscid Isopods underwent some radical modifications of this general pattern [reviewed in Schmalfuss (1998)]. For example the xerophilic desert isopod *Hemilepistus reaumuri* is characterized by a considerable diminution of the first antennae leaving only a very small appendage that is, yet, not without function. Haug and Altner (1984) have shown that in the woodlouse *Porcellio scaber*, the tips of the first antennae are equipped with about 15–20 peg sensilla, which the authors interpret as hygroreceptors, and so called “olfactory hairs” (i.e., aesthetascs) have been reported for *Oniscus asellus* (Ábrahám and Wolsky, 1930). In a behavioral study Zimmer et al. (1996) have demonstrated that *P. scaber* apparently is able to perceive odors and orients toward a food source. However, it is not evident if the animal actually uses antennular olfaction as Hoese and Schneider (1990) stress the importance of constant ground contact of the second antennae while the animal is moving. Nonetheless, a chemoreceptive function of the first antennae of terrestrial isopods cannot be ruled out.

### Deutocerebral chemosensory lobes

The evolutionary size reduction of the first pair of antennae in terrestrial isopods has decreased the sensory input to the DC which resulted in greatly dimished deutocerebral brain areas, and left in most cases not a single trace of neither chemosensory nor mechanosensory areas. Gräber (1933) analyzed the brains of *O. asellus*, *P. scaber*, and *Armadillidium cinereum*, and was not able to identify particular deutocerebral neuropils. Walker (1935) describes a small, untextured neuropil in the ventrolateral DC of *O. assellus* that he considers an olfactory lobe, although such function has not yet been confirmed in behavioral essays. A recent study on the brains of *P. scaber*, *A. vulgare*, and *H. reaumuri* again corroborates the general scheme that the DC in terrestrial isopods is considerably minimized in comparison to aquatic isopods (Harzsch et al., 2011).

In *S. entomon*, at least two neuropils that are associated with the first antenna are present and are comparable to what is found in other malacostracans. The ovoid DCL (i.e., the olfactory lobe) consists of a peripheral array of about 80 radially arranged synaptic spherical fields surrounding a cN of neuronal processes comprising the neurites of local interneurons and projection neurons. These og are subdivided into two distinct layers, a distal cap and a proximal base. For other malacostracans it has been shown that this division mirrors a functional segregation as it is the result of a regionalized innervation pattern of local inter- and projection neurons (Schmidt and Ache, 1997; Schachtner et al., 2005; Polanska et al., 2012). The glomerular organization of DCL as exemplified in *S. entomon* is found in many other malacostracans although variations of this scheme exist. It also has already been regarded to be part of the malacostracan ground pattern (Kenning et al., 2013). In the Decapoda for example, the neuropil experienced several modifications with respect to the general morphology and neuronal architecture of its compartments, in terrestrialized species in particular. In the giant robber crab *Birgus latro*, a terrestrial anomuran, the neuropil is a multilobed complex of more than 1000 markedly elongated, cylindrical og, constituting for roughly half of the total brain volume (Krieger et al., 2010). Moreover, in reptant decapods the glomeruli gain an additional third layer, the subcap, that is located between the cap and base and receives inputs from local olfactory interneurons (see, e.g., Schachtner et al., 2005; Harzsch and Hansson, 2008; Krieger et al., 2010, 2012). This subcap is connected with a neuropil that is only known from reptant decapods, the deutocerebral accessory lobes (Polanska et al., 2012; Sandeman et al., in press). This intricate organization of several deutocerebral neuropils indicates rather sophisticated olfactory systems. As pointed out, our knowledge on the olfactory pathway in Crustacea is mainly founded on studies on Decapoda. Thus, only little information is available for other representatives of the Malacostraca, specifically the Peracarida. Concerning isopods, Harzsch et al. (2011) analyzed various terrestrial representatives but also included a single marine species in the study, the closely related valviferid *I. baltica*. Like *S. entomon*, *I. baltica* possesses aesthetasc bearing first antennae that are associated with a DC housing glomerular DCL. Gräber (1933) examined two species of *Gammarus* (Amphipoda), in both of which, *G. pulex* and *G. fluviatilis* he found rather large DCL housing glomeruli. Within Malacostraca, a similar organization is found in the brains of Stomatopoda (Derby et al., 2003), Euphausiacea (Johansson and Hallberg, 1992), and even Phyllocarida (Kenning et al., 2013), all of which possess DCL that are build up of spherical og, though much fewer in numbers than in Decapoda.

### Projection neuron tract

The PNT, relaying information from the DCL to higher-order processing areas in the lPC (Sandeman et al., 1992; Sullivan and Beltz, 2004), has sparked many discussions as it potentially is of phylogenetic significance concerning the relationships of insects and crustaceans (Strausfeld, 2012). A decussation of this tract in Isopoda has long been negated, although this feature is regarded as a character that was already present in the ground pattern of the Malacostraca (Kenning et al., 2013). According to Hanström (1968), the PNT in the litoral species *Ligia occidentalis* interconnects only ipsilateral neuropils. Equally, in true terrestrial isopods (e.g., *O. asellus* and *P. scaber)* a chiasm is reported to be missing (Gräber, 1933; Walker, 1935), whereas in the amphipod *G. pulex* the PNT features a contralateral connection (Gräber, 1933). As this tract also shows a prominent chiasm in *S. entomon*, it is hard to draw a plausible conclusion with only limited possibilities of comparison, not to mention the unresolved phylogeny of the Isopoda. Keeping in mind that a decussation provides an entirely new level of complexity in neuronal integration pathways, it is hardly conceivable that it evolved convergently in different peracarid representatives. Therefore, we believe that the most likely fate of the chiasm was a reduction of contralateral fibers in the oniscid line, probably due to the aforementioned reduction of antennular input that had a cascading effect on associated structures.

### Lateral protocerebrum

Like the DCL, the neuropils of the lPC have received some attention by researchers and have been described in a number of crustacean taxa. The lPC is composed of the medulla terminalis and the HE and especially the latter has been thoroughly investigated as it seem to play a key role in olfactory learning and constitutes the site of olfactory and multimodal integration (Maynard and Dingle, 1963; Maynard, 1965; Maynard and Yager, 1968; Sullivan and Beltz, 2004; Strausfeld, 2012; Loesel et al., 2013). Information on the organization of the lPC in Isopoda or even Peracarida is scarce but some of the figures provided by Harzsch et al. (2011) allow to draw conclusions on the morphology of the neuropils in question. In all three terrestrial taxa investigated, a neuropil reminiscent of a HE seems to be present [see Figures 5, 6 in Harzsch et al. (2011)], contradicting descriptions of Hanström (1968). However, architecture and innervation patterns are unknown. Corresponding neuropils have also been documented in *Mysis relicta* and *Callomysis maculata* (Mysidacea) but instructive descriptions are missing Hanström (1968). Whereas in *S. entomon* the neuropil shows no signs of subdivision, studies on *Coenobita clypeatus* (Anomura) revealed that their HE is intricately structured and features several subdivisions into multiple cap-neuropils mounting a core neuropil. In these, ascending information from both DCL is relayed to a dense, rectilinearly multilayered network (Brown and Wolff, 2012; Polanska et al., 2012; Wolff et al., 2012), and may thus be involved in higher-order olfactory processing.

### Lateral antenna 1 neuropils

In Malacostraca, mechanosensory input from the antenna 1 is relayed to the lateral antenna 1 neuropil. At least for decapods, it has been shown that it also receives afferents from the statocyst and non-aesthetasc chemoreceptors (Sandeman and Denburg, 1976; Yoshino et al., 1983; Roye, 1986; Blaustein et al., 1988; Schmidt and Ache, 1993), and is involved in controlling the movements of the first antenna (Maynard, 1965; Roye and Bashor, 1991; Schmidt and Ache, 1993, 1996). For several representatives of Malacostraca it has been noted that this neuropil shows a horseshoe-like appearance, and Kenning et al. (2013) considered this organization as plesiomorphic for this taxon. However, this trait seems to have been lost in the isopod clade. Although a lateral antenna 1 neuropil is present and likely takes part in controlling the movements of the first antenna, a contribution to the equilibrium sense is unlikely as statocysts in Isopods are located in an anterolateral process of the cephalon at the level of and just anterior to the compound eyes and being associated with a not further specified region in the lPC (Wenig, 1903; Walker, 1935).

### Tritocerebral neuropils

Like in other malacostracans, the second antenna of *S. entomon* relays its input into a comparatively large neuropil area, the antenna 2 neuropil. Yet, here it is of quite conspicuous texture showing a microglomerular organization like it has been reported for *H*. *reaumuri* (Harzsch et al., 2011) and other terrestrial representatives of the Isopoda (Gräber, 1933; Walker, 1935), an aspect that may been overlooked in the study of Harzsch et al. in *A. vulgare* and *P. scaber*. While a glomerular neuropil organization is often associated with a chemoreceptive function (Strausfeld, 2012), it is known from various other malacostracan crustaceans that their antenna 2 neuropils are longitudinally subdivided into repeated units, and receive the somatotopic representation of the chemo- and mechanosensory sensilla on antenna 2 (Tautz and Müller-Tautz, 1983; Zeil et al., 1985; Sandeman and Varju, 1988; Krieger et al., 2012). Interestingly, this is also the case in the marine *I. baltica* (Harzsch et al., 2011). The finding of a microglomerular organization of the antenna 2 neuropil in the closely related *S. entomon* but not an (obvious) longitudinal subdivision raises questions about its sensory significance. As the first antennae are greatly reduced in oniscid isopods, the second antennae are thought to function as the major sensory organs [reviewed in Schmalfuss (1998)] with their unstalked eyes playing a subsidiary role. Similar to their marine relatives, all segments of their second antennal pair carry numerous mechanoreceptive sensilla. In addition, the terminal segment bears a tuft of robust sensilla, the apical organ or apical cone which is interpreted as detecting both mechanical and chemical stimuli (Mead et al., 1976; Alexander, 1977; Hoese, 1989; Hoese and Schneider, 1990; Schmalfuss, 1998). In the desert isopod *H. reaumuri*, the apical organ has even been suggested to respond to both olfactory and gustatory stimuli (Seelinger, 1977, 1983). Although responses to distant olfactory stimuli have not yet been tested behaviorally, their apical organs likely play a key role in the perception of chemical signals involved in social recognition, family cohesion, congregation, and communication (Fischbach, 1954; Kuenen and Nooteboom, 1963; Linsenmair, 2007). To what extend this might also hold true for a marine representative remains uncertain.

### Neuroethological considerations and conclusion

Our findings suggest that *S. entomon* not only has the morphological substrate to perceive but also to process olfactory stimuli. However, without lab-based ethological investigations, statements on the animals' sensory capabilities are pure conjecture. Yet, it may nevertheless be enlightening to discuss previous electrophysiological investigations of the eyes, ultrastructural examinations of the aesthetascs, and our own findings with regard to the animal's life style. *S. entomon* is an opportunistic and omnivorous nocturnal scavenger that lives on dead fish and other carcasses as it may find on the sea bottom but also actively preys on amphipods, conspecifics, and other isopods (Green, 1957; Haahtela, 1990; Leonardson, 1991). Whereas younger populations of *S. entomon* migrate into shallow water coastal areas during the winter, the main depth of occurrence is 50–85 m and even depths down to 290 m have been reported. The eyes show a relatively high sensitivity with maximum absorption close to the wavelength of maximum light transmittance in their habitat waters (Lindström et al., 1991). However, the dorsal position of the eyes in *S. entomon* and low light levels in its habitat raises the question if this animal's feeding behavior is mostly visually guided or rather if other senses are substantial for the survival of this species. Preliminary experiments by Pynnönen (1985) suggest that *S. entomon* is chemically attracted to food sources and that the aesthetasc equipment of antenna 1 is necessary for a directed movement toward a food source. Observations in the field as well as in the laboratory indicate that *S. entomon* does react and orients toward dead fish and fluid homogenates of conspecifics but that tracking movement, however, is fairly undirected and random (personal observations by the authors). This requires further investigations in bioassays to evaluate which role chemical stimuli play for the feeding behavior. A corresponding experiment has been conducted using *Scopelocheirus onagawae* (Amphipoda) that demonstrated nicely how the animals react to and congregate at agar blocks saturated with a certain synthetic amino acid mixture, imitating rotting fish (Ide et al., 2006).

The evidence discussed above demonstrates that in Isopoda a significant reconfiguration of appendages and brain areas involved in olfaction has taken place during the evolutionary conquest of land. Along these lines, it may be well worth exploring which other adaptations the Isopoda might have evolved in their visual, chemo- and mechanosensory systems when considering the dramatically different sensory ecology, e.g., in a challenging habitat like the abyssal plains or in animals with more derived life styles such as parasitic isopods.

## Author contributions

Matthes Kenning designed and performed the experiments, and analyzed the data under supervision of Steffen Harzsch. Both authors wrote the manuscript, discussed the results and implications and commented on the manuscript at all stages.

### Conflict of interest statement

The authors declare that the research was conducted in the absence of any commercial or financial relationships that could be construed as a potential conflict of interest.

## Abbreviations

(n): cellcluster
a: anterior
A1Nv: antenna 1 nerve
A2Nv: antenna 2 nerve
ae: aesthetasc
AnN: neuropil of antenna 2
ao: apical organ
cA: “chemosensory” afferents
CB: central body
cN: coarse neuropil
d: dorsal
iCh?: presumptive inner optic chiasm
HE: hemiellipsoid body
DC: deutocerebrum
DCL: deutocerebral chemosensory lobe
DGN: dorsal giant neuron
fl: flagellum
l: lateral
La: lamina
lal: lateral accessory lobe
LAN: lateral antenna 1 neuropil
LNv: labral nerve
lF: lateral foramen
Lo: lobula
LoP: lobula plate
lPC: lateral protocerebrum
MAN?: presumptive median antenna 1 neuropil
MdC: mandibular commissure
MdNv: mandibular nerve
Me: medulla
mA: “mechanosensory” afferents
mF: median foramen
mPC: median protocerebrum
MT: medulla terminalis
oc: esophageal connective
oCh: outer optic chiasm
oe: esophageal foramen
og: olfactory glomeruli
oNv: optic nerve
optN: optic neuropils
PC: protocerebrum
PCC: protocerebral commissure
PB: protocerebral bridge
pe: peduncle
PNT: projection neuron tract
PT: protocerebral tract
R: retina
TC: tritocerebrum
TNv: tegumentary nerve
vC: visceral commissure
VNC: ventral nerve cord
W: X, Y, Z see text.
